# Impact of Knowledge Hiding Behaviors on Workplace Invincibility: Mediating Role of Psychological Contract Breach

**DOI:** 10.3389/fpsyg.2021.809683

**Published:** 2022-01-25

**Authors:** Junqi Wen, Jiafeng Zheng, Ruijun Ma

**Affiliations:** ^1^School of Labor and Human Resources, Renmin University of China, Beijing, China; ^2^Graduate School of Architecture, Planning and Preservation, Columbia University, New York, NY, United States

**Keywords:** knowledge hiding, evasive hiding, playing dumb, rationalized hiding, psychological contract breach, workplace incivility

## Abstract

In recent years, knowledge hiding has gained much popularity in the knowledge management literature. Apart from that, antecedents and consequences of knowledge hiding are being scrutinized at present. There have been many studies on the triggering forces of knowledge hiding; however, the uncivil behaviors at the workplace have led the organizations and employees in trouble due to its possible associating factors, which is well explained by social influence theory. One such factor that this study has identified is knowledge hiding behavior at the workplace. This is a quantitative cross-sectional study based on a survey. The population taken in this study is the middle and low-level managerial staff of the software houses located in China. The respondents were selected based on convenient random sampling, and a sample size of 287 is used in this study. The data collected were employed with the partial least square structural equation modeling using Smart-PLS 3. The findings of this study show that apart from evasive hiding, playing dumb and rationalized hiding plays a significant role in predicting workplace incivility. In addition, psychological contract breach (PCB) has been taken as the mediating variable. The violation of psychological contracts among employees can indulge them in negative feelings that may convert to workplace incivility at any available opportunity of revenge which is well explained by social influence theory. Workplace incivility cannot be completely eradicated from the organizations; however, it can be controlled by making relevant policies. The civility among the employees can be attained by due managerial interventions and training of the employees considering the protection of victims and due punishment to the perpetrator.

## Introduction

Knowledge management is a valuable asset for individuals and organizations, which usually has no substitute and may act as a strategic asset to gain a competitive advantage (Connelly and Zweig, 2015; Issac et al., 2021). Knowledge hiding has been the topic of interest for researchers in an organizational context as it has led to many favorable (such as creativity and organizational performance) and unfavorable (interpersonal distrust, lack of career growth, and competition) outcomes which may either constrain or foster the process of productivity, effectiveness, and growth depending upon the intentions of knowledge hider (Khoreva and Wechtler, 2020). It has been defined as the intentional concealment of a requested piece of knowledge (Guo et al., 2021). Recent literature on knowledge hiding has been considered to limit the useful knowledge transfer, and negative spiral reprisal arises as knowledge sharing is not as easy as said by Èerne et al. (2014), Connelly et al. (2019), and Hernaus et al. (2019). Hence, Issac et al. (2021) have declared knowledge hiding as a counterproductive behavior at the workplace, leading to unfavorable and devastating outcomes that are not intended to harm but unacceptable if exposed. One such outcome is an effective organizational performance that is crucial in this era of the knowledge economy.

Literature has been reporting such unfavorable behaviors of employees in organizations for many reasons such as busy schedules, lack of time, workload, which restrain people from being good to others (Boz Semerci, 2019). Such behaviors that have been found frequently among employees in different organizations include doing something else during meetings, impolite behavior, being rude and discourteous, social undermining, personal attacking, degrading others, deception, etc. (Arshad and Ismail, 2018; Irum et al., 2020). Researchers have called these discounted negative behaviors as uncivil behavior and termed it in organizations as workplace incivility (Aljawarneh and Atan, 2018; Akella and Lewis, 2019; Alias et al., 2020; Chughtai et al., 2020; Megeirhi et al., 2020; Vasconcelos, 2020). However, these behaviors go unnoticed because of low intensity, minimum negative outcome, and ambiguous intentions to harm others (Arshad and Ismail, 2018), and these deleterious behaviors have been ignored in the past that have led to certain outcomes such as team conflict, knowledge hiding, competition, interpersonal distrust, workplace aggression, lack of tolerance (Taylor and Kluemper, 2012; Aljawarneh and Atan, 2018; Arshad and Ismail, 2018; Strik et al., 2019; Issac et al., 2021; Karim et al., 2021; Sheidae et al., 2021).

People interact with each other based on exchange and mutual expectations of giving and taking favors, which is usually explained by social exchange theory (SET). The employees in the organizations act according to the SET that explains the behavior of giving favors to other individuals based on the expectation of getting favors in return. However, it does not always happen. When an individual expects certain favors in return and organization and individuals do not abide by this unsaid and psychological contract of returning a favor, negative emotions for each other arise, and a psychological contract breach (PCB) happens (Pervez et al., 2019). According to Bari et al. (2020), when the employees in an organization do not meet their due favors to others, the psychological contract is a breach. Such negative emotions or the breach of any psychological contract may lead to workplace incivility in the organizations. Hence, it is important to understand the roles of knowledge hiding and PCB in workplace incivility.

The main objective of this study is to understand how knowledge hiding and workplace incivility are correlated in the organizational setup using social influence theory in the presence of PCB. Knowledge hiding and workplace incivility have not been incorporated with the mediating role of PCB, which is a serious gap in the literature of knowledge hiding. Therefore, this study measures the role of knowledge hiding in workplace incivility in the presence of PCBs in organizations.

## Review of Literature

### Knowledge Hiding and Workplace Incivility

Knowledge management has been a critical asset for individuals and organizations that give a competitive advantage at the time of complexity, volatility, ambiguity, and uncertainty. Knowledge can improve organizational performance and innovation when utilized adequately (Pradhan et al., 2020). Mostly in organizations, employees are motivated and encouraged to share knowledge, either tacit or explicit, and spend a huge amount of money on upgrading their knowledge management systems and creating a conducive environment for facilitating the knowledge sharing in the organization (Jahanzeb et al., 2020). However, when negative reciprocity has been seen in the organizations, employees are motivated negatively and react unacceptably. Hence, when individuals experience negative or abusive behavior from their supervisors or fellows, they indulge in counterproductive behaviors at the workplace; knowledge hiding is one such behavior which is seen mostly in organizations (Ghani et al., 2020).

In 2006, the survey of Globe-Mail about 1,700 readers, 76% of them were found to engage in the knowledge hiding behavior (Aljawarneh and Atan, 2018). In the literature, knowledge sharing and knowledge hiding have been considered as the opposite concepts to each other; however, these two concepts are found to have distinct motives, circumstances, and contexts (Pradhan et al., 2020). According to Baig and Waheed (2016), knowledge hiding is not the mere lack of knowledge sharing but it has been considered as a negative behavior where the individuals intentionally withhold their knowledge (Connelly et al., 2012). According to Anand et al. (2020), knowledge hiding is that which shares the piece of knowledge with others to a certain extent, which has the deliberate intention of sharer involved in not sharing it with the seeker. Knowledge hiding has been defined in the literature as the intentional effort for not sharing the knowledge or concealing it from those who requested it (Hernaus et al., 2019). There have been many reasons that the employees do not share knowledge with others, especially if it is related to their job, new ideas, or most sought information. There has been a piece of clear evidence in the literature that secrecy and the psychological ownership of the knowledge are the fundamental reasons for the knowledge hiding seen in organizations (Pierce and Dirks, 2001; Karahanna et al., 2015; Dawkins et al., 2017; Zhu et al., 2018; Ghani et al., 2020).

Furthermore, when there is a scene of competition and career growth, employees tend to hide their top understanding of knowledge hidden (Issac and Baral, 2018). However, some authors have argued that knowledge hiding cannot always be harmful. Sometimes, it is intended for some hidden benefits (Abe et al., 2014; Toma et al., 2016) as confidentiality and protecting their or any other favorite party’s interests. The hidden knowledge can have certain motives associated with it, which can be internal or external to individuals and organizations (Anand et al., 2020).

According to Connelly et al. (2012), knowledge hiding occurs only when a request for knowledge is not fulfilled. However, there are different ways of not sharing the knowledge with the seeker. Therefore, the literature has divided knowledge hiding into three types (Connelly et al., 2012; Zhao et al., 2016; Kumar Jha and Varkkey, 2018; Bari et al., 2019, 2020; Hernaus et al., 2019; Samdani et al., 2019; Irum et al., 2020; Khoreva and Wechtler, 2020; Ma et al., 2020).

(a) *Evasive hiding*

Evasive hiding has been defined as a type of knowledge hiding where the individual does not share knowledge with others but rather ensures them that the requested piece of knowledge will be shared later.

(b) *Playing dumb*

Playing dumb is the strategy for knowledge hiding where the individual refuses to share the knowledge or denies having any of the requested knowledge.

(c) *Rationalized hiding*

Rationalized knowledge hiding is the type of knowledge hiding where the sharer does not share the requested knowledge, justifying his/her behavior for not sharing. The sharer does not conceal the piece of knowledge rather gives explanations why he is not sharing the knowledge.

Despite much types of research on antecedents of knowledge hiding, managers have not yet been able to control this knowledge hiding behavior in organizations (Anand et al., 2020). Though there have been few types of research about the consequences of knowledge hiding (Zhao et al., 2016; Labafi et al., 2017; Kumar Jha and Varkkey, 2018; Bari et al., 2019; Butt and Ahmad, 2019; Iqbal et al., 2020; Alnaimi and Rjoub, 2021; Zhai et al., 2021), those are not enough to understand the wholesome contexts for knowledge hiding’s unfavorable consequent behaviors. However, the consequences of knowledge hiding need the researcher’s attention. Since knowledge hiding may create a negative atmosphere among the employees that drives them to behave negatively or keep grudges about colleagues, which creates an aura of mutual distrust or violation of the psychological contract among them, disclosure of knowledge hiding or such a negative atmosphere may trigger the negative behavior of employees with each other which causes workplace incivility.

Workplace incivility is considered a disruptive behavior in organizations that disturbs the daily social phenomenon among the employees (Alias et al., 2020). Individuals in the organizations have certain expectations with their colleagues and the employer; one of those expectations is to be treated fairly and justly along with the fulfillment of the promises made at the time of hiring and induction (Pradhan et al., 2020). However, when the employees are not treated according to their expectations, they consider it the betrayal at the employer’s part; they become aggrieved and considered abuse and develop negative feelings. They are triggered by these negative feelings and consider the organization as the main culprit for not taking due action for this unjust behavior (Jahanzeb et al., 2020). Therefore, the employees develop feelings of counterproductivity, uncivil attitude, and the feeling of resentment at the workplace. Workplace incivility is the maltreatment of individuals with others; however, such behaviors are not intense and are not intended to cause any serious harm (Chughtai et al., 2020). In the past researches, workplace incivility has been related to psychological and health-related outcomes (Cortina and Magley, 2009), job stress, interpersonal deviance (Blau and Andersson, 2005), mental health, aggression (Lim et al., 2008, 2016), personality traits (Milamm et al., 2009), task interdependence (Ghosh et al., 2011), mental disorders (Gedro and Wang, 2013), psychological contract violation (Sears and Humiston, 2015), emotional exhaustion, Moon and Hur (2018), and psychological strain (Mullen and Kelloway, 2013) as the antecedent or consequent of these variables. It has been argued as an antisocial behavior which includes low intense harms such as anger, aggression, deviance, bullying, and workplace violence (Chughtai et al., 2020). These malicious behaviors can be exhibited due to any negative trigger such as knowledge hiding or interpersonal distrust or breach of psychological contract (Agarwal and Narayana, 2020).

There have been different definitions to workplace incivility. Megeirhi et al. (2020) have defined workplace incivility as the negative feelings of coworkers that harm interpersonal relationships. Workplace incivility is different from other bullying behaviors in organizations as it is lowest in intensity and has minimum intention to harm others (Aljawarneh and Atan, 2018). It has been stressed as the outcome of some unwanted behaviors such as knowledge hiding. Akella and Lewis (2019) have argued that the changes in the organizational structure and policies are considered the leading causes of workplace incivility. Organizational factors such as downsizing, vacancies for new inductions, mergers and acquisitions, shifts in human resource policies, restructuring, alternative working shifts, and altered psychological contracts among employees are the sources of workplace incivility in the organizations. Other forms of workplace incivility are based on differences in gender, age, and race and the interpersonal differences that arise over time (Blau and Andersson, 2005; Akella and Lewis, 2019). Since many triggering factors have been reported in the previous literature, knowledge hiding is another reason for workplace incivility. Though substantial literature is available on workplace incivility, the role of knowledge hiding as the driving factor remains unnoticed. Therefore, this study has made the following hypotheses based on the three types of knowledge hiding available in the literature:

***H_1_:*** *Evasive hiding has a positive association with workplace incivility.*

***H_2_:*** *Playing dumb has a positive association with workplace incivility.*

***H_3_:*** *Rationalized hiding has a positive association with workplace incivility.*

### Psychological Contract Breach as Mediator

Psychological contract breach has been the variable of interest for researchers for many years to explain the social interactions using SET. People make connections and interact with others in the hope that the favor they are doing to others will be returned at some time in future (Rani et al., 2018; Pervez et al., 2019; Agarwal and Narayana, 2020; Bari et al., 2020; Han et al., 2021). The social relationships among the employees are naturally based on the social exchanges, an expectation of getting a reciprocal favor in the future for a good deed done at present (Zhai et al., 2021). When these behaviors are not reciprocated as expected, employees indulge in PCBs. Individuals with high psychological ownership are seen to react more intensely than those who have less psychological ownership (Karahanna et al., 2015; Dawkins et al., 2017; Zhu et al., 2018). Formally, it has been defined as the perception of employees that they have not received back the favors and privileges from the employer or colleagues that were informally or formally promised (Ghani et al., 2020). Psychological contract breach has been established in the literature as the failure of either employee or employer (in case of an organization) or either party in fulfilling their duties toward the other (Rani et al., 2018).

Knowledge hiding can significantly damage the interpersonal relationships in the organizations by creating an atmosphere of distrust among employees and ultimately reducing the organization’s productivity and performance (Hernaus et al., 2019). The practices of knowledge hiding have been found common in the organizations when sharing the said piece of information with others; it is believed that some part of knowledge is kept hidden (Pradhan et al., 2020). Most of the studies have focused on PCB concerning cronyism and moral disengagement (Pervez et al., 2019), trust and distrust (Rani et al., 2018), and knowledge hiding (Bari et al., 2020); however, less is known for its role with workplace incivility. Henceforth, current research contributes to the body of literature regarding PCBs by measuring the underlying mechanism of PCB and its role in workplace incivility.

A psychological contract creates a mutual strategic relationship between the parties to collaborate and maintain an adequate and better understanding of their working affiliations (Rani et al., 2018). Employees working in an organization or the individuals working in a team develop certain expectations toward each other and they feel indebted when one party gives the other a favor. They get more involved and put more effort into their tasks and expect the other individuals and/or organizations to keep treating them fairly (Robinson and Morrison, 2000; Alcover et al., 2017; Dawkins et al., 2017; Rani et al., 2018; Zhu et al., 2018; Pervez et al., 2019; Khalid et al., 2020; Menec et al., 2020). However, when one party does not meet the expectations of others, a breach of psychological contract happens. Thus, it defines the implicit and explicit duties of the employee and the employer toward each other. Fundamentally, a PCB refers to the annoyance caused by the disturbance of the fulfillment of the promises on behalf of the organization or any fellow (Rani et al., 2018).

The employees of an organization are usually perceived as the representatives of their respective organizations, and any unjust or unfair act of them is taken as unacceptable from their organization (Qian et al., 2021). Therefore, when the employee at the top deals unfairly the rest of the employees and favors their friends or favorites, unseen PCB happens, which leads to unacceptable behaviors that are counted for workplace incivility (Pervez et al., 2019). Such individuals existing in the organization negatively affect the organizational environment (Bari et al., 2020) that indulges the victims of knowledge hiding into workplace uncivil behaviors. Hence, based on the literature above, the following hypotheses have been formulated considering the mediating role of PCB in the relationship between knowledge hiding and workplace incivility.

***H_4_:*** *PCB mediated the positive association between evasive hiding and workplace incivility.*

***H_5_:*** *PCB mediated the positive association between playing dumb and workplace incivility.*

***H_6_:*** *PCB mediated the positive association between rationalized hiding and workplace incivility.*

## Methodology

**FIGURE 1:**
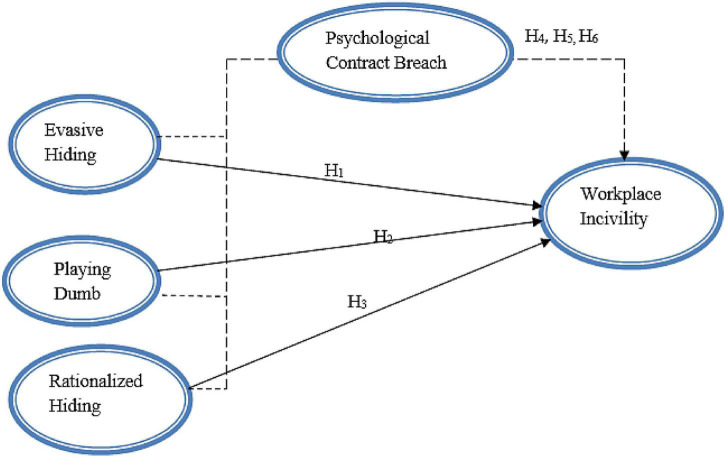
Theoretical framework.

Based on the gaps found in the literature, following relationships of the variables have been proposed in the theoretical framework as shown in Figure 1. Evasive hiding, playing dumb, and rational hiding are three types of knowledge hiding proposed to contribute to workplace incivility with PCB mediating these relationships. Below framework is based on social influence theory that states that the behaviors of individuals are affected by the behaviors of others.

## Research Methodology

In this study, the three types of knowledge hiding, that is, evasive hiding, paying dumb, and rationalized hiding have been checked for their association with workplace incivility and the mediating role of PCB. Since this study is about checking the effect of knowledge hiding subvariables on workplace incivility, hence the philosophy followed is postpositivism. The research approach followed is the deductive approach as the theories are proposed through the hypotheses and then are checked through the quantitative techniques for data analysis. To collect the data for this cross-sectional study, a structured questionnaire was used. The questionnaire was divided into two sections. The first section was about the demographics of the respondents, whereas the second part contained the items that measure the variables of the study. The population for the study used was the middle and lower management staff of the software houses. A total of 287 responses were received based on convenient random sampling considering the convenient access of the authors to the sample. The results of the demographic questions are shown in Table 1.

**TABLE 1:** Demographics of the respondents.

Summary	Frequency	%
**Gender**		
Male	161	56.09
Female	126	43.90
**Age**		
<25	121	42.16
25–30	56	19.51
31–40	35	12.19
41–50	20	6.96
>50	55	19.16
**Education**		
Higher secondary	59	20.55
Bachelor	104	36.23
Masters	80	27.87
Doctorate	44	15.33
**Designation**		
Manager	158	55.05
Assistant	129	44.94

*N = 287.*

### Instrument Development

There are total of five variables in this study; three independent variables, that is, evasive hiding, playing dumb, and rationalized hiding; one mediating variable which is PCB and one dependent variable which is workplace incivility. The measurement scale used in this study was used in previous studies; however, they were tested for reliability and validity in this study. The scale was developed using the measurement scales used in the previous studies for measuring the respective variables. The scale of three types of knowledge hiding, that is, evasive hiding, playing dumb, and rationalized hiding has been adapted from the study by Connelly and Zweig (2015), PCB from study by Robinson and Morrison (2000), and workplace incivility from study by Di Marco et al. (2018). The scale of three variables addressing knowledge hiding contained 4 items each, the PCB was measured with 5 items scale, and workplace incivility was measured with 4 items scale. The questionnaire used in this study was developed on a five-point Likert scale with 1 = strongly disagree and 5 = strongly agree.

## Data Analysis

The data analysis in this study is performed in three stages. First of all, the demographic characteristics have been analyzed, and then the measurement model was checked for the validities and reliability of the data and how the variables are measured. In the third stage, the structural model is analyzed for the relationship of the hypotheses. The data analysis in this study is performed through Smart-PLS which utilized the partial least square structural equation modeling.

In the first stage, the demographic characteristics of the respondents were analyzed using the frequencies and percentages for the respondents’ responses. The demographic questions included in the study were related to the respondents’ age, gender, education, and managerial level. The results can be seen in Table 1.

**FIGURE 2:**
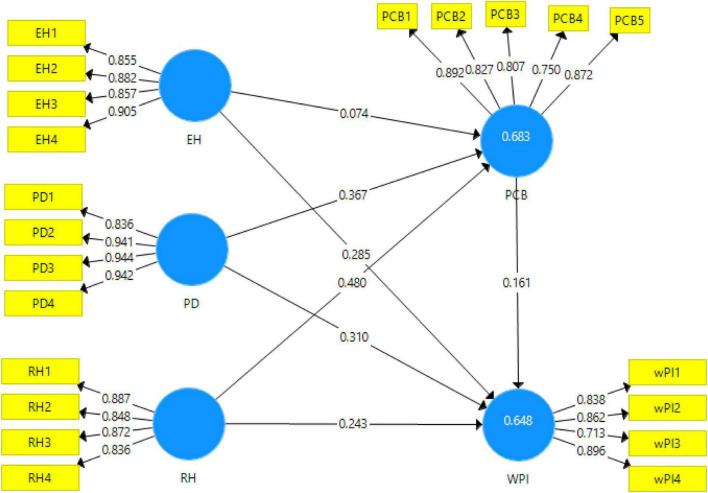
Measurement model algorithm.

The measurement model is obtained as the first stage of Smart-PLS is run (Figure 2). It shows the reliabilities and the validities of the scales used. In this study, the results obtained from the measurement model have been reported in Table 2.

**TABLE 2:** Constructs reliabilities and AVE.

Constructs	Code	FD	*α*	CR	AVE
Evasive hiding			**0.899**	**0.929**	**0.766**
	EH1	0.855			
	EH2	0.882			
	EH3	0.857			
	EH4	0.905			
Playing dumb			**0.936**	**0.955**	**0.841**
	PD1	0.836			
	PD2	0.941			
	PD3	0.944			
	PD4	0.942			
Rationalized hiding			**0.883**	**0.920**	**0.741**
	RH1	0.887			
	RH2	0.848			
	RH3	0.872			
	RH4	0.836			
Psychological contract breach			**0.887**	**0.918**	**0.691**
	PCB1	0.892			
	PCB2	0.827			
	PCB3	0.807			
	PCB4	0.750			
	PCB5	0.872			
Workplace incivility			**0.847**	**0.897**	**0.688**
	WI1	0.838			
	WI2	0.862			
	WI3	0.713			
	WI4	0.896			

*N = 287, FD = factor loading, CR = composite reliability, AVE = average variance extracted, α = Cronbach’s alpha reliability.*

The factor loadings for the items used for each variable showed the acceptable ranges; that is, it should be more than 0.7 (Avotra et al., 2021; Nawaz et al., 2021). All the values obtained in factor loadings are above the cutoff value. Alpha reliabilities obtained are also according to the set criteria that it should be close to 1. The values obtained ranged from 0.847 to 0.936, which meet the criteria for alpha reliability (Qin et al., 2021; Yingfei et al., 2021). Moreover, the cutoff value mentioned in the literature for the average variance extracted is 0.5 (An et al., 2021). The values obtained from the measurement model in the study are all above the said criteria, hence making the variables valid. Further validation of the data was checked through Fornell and Larcker criterion and heterotrait-monotrait (HTMT) ratio. The algorithm obtained from the measurement model is given below.

Furthermore, the convergent and discriminant validities were checked for the data obtained in the study. In this study, the discriminant validity is measured through the Fornell and Larcker criterion and HTMT ratio. The results for these results are given in Tables 3, 4, respectively.

**TABLE 3:** HTMT ratio.

	EH	PCB	PD	RH	WPI
EH	**0.875**				
PCB	0.345	**0.831**			
PD	0.308	0.761	**0.907**		
RH	0.327	0.788	0.772	**0.861**	
WPI	0.515	0.687	0.708	0.703	**0.830**

*N = 287, EH = evasive hiding, PCB = psychological contract breach, PD = playing dumb, RH = rationalized hiding, WPI = workplace incivility. Bold values mean the significance between variables.*

**TABLE 4:** Fornell and Larcker criterion.

	EH	PCB	PD	RH	WPI
EH					
PCB	0.379				
PD	0.329	0.829			
RH	0.357	0.888	0.840		
WPI	0.598	0.781	0.771	0.799	

*N = 287, EH = evasive hiding, PCB = psychological contract breach, PD = playing dumb, RH = rationalized hiding, WPI = workplace incivility.*

The results obtained from Fornell and Larcker test show that the values at the top of each column are the highest, which show the significance of the Fornell and Larcker criterion for the discriminant validity of the data. In this study, the evasive hiding column has a value of 0.875, which is the highest among all values that are at the top of that column. Similarly, for PCB, 0.831 is the highest, and so on.

The HTMT ratio obtained should be below 0.9 for the data to be valid (Nawaz et al., 2020). The values obtained in this study are below 0.9, and the highest value in the table is 0.840 which meets the criterion for the discriminant validity in this study. The results obtained for the HTMT ratio are reported in Table 4.

**FIGURE 3:**
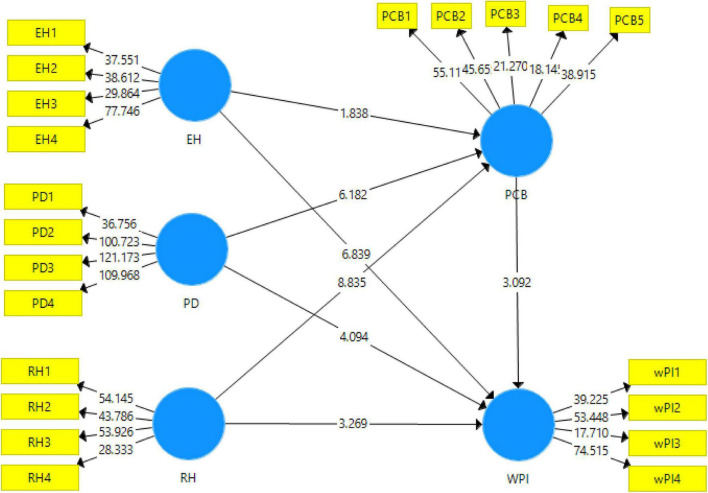
Structural model algorithm.

The structural model obtained from the bootstrapping using Smart-PLS has been reported in the Table 4 along with the structural model algorithm that is shown in the Figure 3.

Data analysis for the measurement of the variables has been performed using structural equation modeling (SEM) with the technique of partial least square (PLS). The results obtained from the structural model algorithm using Smart-PLS have been reported in Table 5. This study has used beta values, *t*-statistics, *f*-square values, *r*-square values, and *p*-values at 5% significance level to accept or reject the hypotheses based on the direction of relationships, the strength of relationships, and effect sizes. The association of the evasive hiding with workplace incivility as mentioned in H_1_ has been rejected at 5% level of significance with beta = 0.285, *t*-statistic = 1.912, *p*-value = 0.057 at the borderline. For the second and third hypotheses that addressing the association of playing dumb (beta = 0.310, *t*-statistic 6.272, *p*-value = 0.000) and rationalized hiding (beta = 0.243, *t*-statistic = 8.784, *p*-value = 0.000) have been found significant, hence H_2_ and H_3_ are accepted. Similarly, the PCB mediation has been checked with the three types of knowledge hiding. First mediation with evasive hiding has been rejected (*t*-statistic = 1.359, *p*-value = 0.175). The second mediation with playing dumb (*t*-statistic = 2.818, *p*-value = 0.005) and rationalized hiding (*t*-statistic = 2.933, *p*-value = 0.004) has been accepted based on 95% confidence interval. These accepted hypotheses (H_2_, H_3_, H_5_, and H_6_) have shown the positive association of playing dumb and rationalized hiding with the workplace incivility and positive mediation of PCB for the said independent variables. Moreover, workplace incivility and PCB have been found strong variables which are predicted 64.8% and 68.3% by evasive hiding, playing dumb, and rationalized hiding.

**TABLE 5:** Results for structural model.

Paths	H	Path coefficients	*f*-square	*t-* statistics	*p*- values	R^2^	Results
EH **→** WPI	H1	0.285	0.201	1.912	0.057	0.683	Rejected
PD **→** WPI	H2	0.310	0.094	6.272	0.000***		Accepted
RH **→** WPI	H3	0.243	0.052	8.784	0.000***		Accepted
EH **→** PCB **→** WPI	H4	0.012	0.201	1.359	0.175	0.648	Rejected
PD **→** PCB **→** WPI	H5	0.059	0.171	2.818	0.005**		Accepted
RH **→** PCB **→** WPI	H6	0.077	0.288	2.933	0.004**		Accepted

N = 287, H = hypotheses, ***p < 0.0005, **p < 0.005, EH = evasive hiding, PD = playing dumb, RH = rationalized hiding, PCB = psychological contract breach, WPI = workplace incivility.

## Discussion

Determining the role of knowledge hiding in workplace incivility in the organizations is supposed to give a new direction to the prevailing unacceptable behaviors of the employees. Moreover, understanding the underlying reasons for such behaviors is critical to a friendly and progressive atmosphere at the workplace, which would raise a professional environment, satisfies customers, and improves overall organizational performance. Considering the literature on knowledge hiding, its driving factors, and consequences, this study has identified one of the most prevailing behaviors in organizations that need the researcher’s attention which is workplace incivility; its triggering factors that make indulging in such behaviors justified for some employees. In this study, the role of three main types of knowledge hiding behaviors, namely, evasive hiding, playing dumb, and rationalized hiding has been checked for their contribution to workplace incivility. Further, PCB has been found a significant factor among the employees that encourages workplace incivility to be a just behavior. In this study, the results obtained from Fornell and Larcker test were found significant and showed that the values at the top of each column were highest which confirms the significance of the Fornell and Larcker criterion for the discriminant validity of the data. Furthermore, the evasive hiding column has the value of 0.875, which is the highest value at the top of the respective column. Similarly, for PCB, 0.831 is the highest, and so on. The HTMT ratios obtained were below 0.9 (Franke and Sarstedt, 2019); hence, the data were found valid. The values obtained for the HTMT ratios are below 0.9 and the highest value in the table is 0.840 which meets the criterion for the discriminant validity.

Regarding the first hypothesis of the study (H_1_: evasive hiding is positively associated with workplace incivility), it could not find a significant association with workplace incivility because employees in the organizations do not consider it unethical; however, the sharing of the knowledge is ensured in the future, and hence, no intentional harm in the form of workplace incivility is experienced. On the other hand, regarding the second hypothesis (H_2_: playing dumb is positively associated with workplace incivility) and third hypothesis (H_3_: rationalized hiding is positively associated with workplace incivility), playing dumb and rationalized hiding have been found to have a strong positive association with the workplace incivility, and these results are following the past research conducted by checking the role of workplace incivility in knowledge hiding (Aljawarneh and Atan, 2018; Arshad and Ismail, 2018). This is because when an individual knows that his/her colleague is deliberately hiding the knowledge, it creates grudges for them and workplace incivility is exhibited at any possible opportunity. However, these uncivil behaviors at the workplace are not intense and subjected to cause any harm to fellows.

Moving forward to the mediating variables of the study, there were three mediations that were proposed to be checked for this study. The mediating variable taken in this study is PCB. The hypotheses proposed were the following: H_4_: PCB mediates the positive association between evasive hiding and workplace incivility, H_5_: PCB mediates the positive association between playing dumb and workplace incivility, and H_6_: PCB mediates the positive association between rationalized hiding and workplace incivility. Regarding the hypothesis H_4_, evasive hiding could not be associated with workplace incivility *via* the mediation of PCB because evasive did not openly show the negative feeling because this is not the refusal or denial of sharing knowledge with others; therefore, no breach of psychological contract is considered from the other party. However, regarding H_5_ and H_6_, association of playing dumb and rationalized hiding with workplace incivility was significantly mediated by PCB. PCB has been considered as a strong mediator in organizational behavior which is also supported by past researches (Robinson and Morrison, 2000; Alcover et al., 2017; Dawkins et al., 2017; Rani et al., 2018; Zhu et al., 2018; Khalid et al., 2020; Menec et al., 2020). It is because when knowledge is hidden and the colleagues or the employee realizes it, they start to develop negative feelings for the counterpart, therefore getting indulged in the malicious activities that are called workplace incivility in management literature.

## Theoretical Contributions

Following are the theoretical contributions made in the literature. (1) It has been found that apart from evasive hiding, playing dumb and rationalized hiding play a significant role in predicting the workplace incivility directly and also in the presence of PCB among the parties. It has been justified with the help of social influence theory in which the behaviors of some people are influenced. (2) The breach or violation of the psychological contract among the employees creates the aura of competition and negative vibes with the employer for not taking due actions and treating justly. Hence, the presence of PCB gets the employees involved in workplace incivility that has been studied in this study.

### Managerial Implications

(1) Workplace incivility cannot be completely eradicated from the organizations (Aljawarneh and Atan, 2018); however, it can be controlled by making relevant policies. (2) This study helps the organizations in devising the strategies following the possibility of knowledge hiding among employees. (3) As this study has found workplace incivility as one of its consequences, possible policies should be devised considering the protection of victims and due punishment to the perpetrator. (4) Moreover, there should be a proper code of conduct regarding workplace incivility that creates awareness among the employees and detains them from becoming victims. The civility among the employees can be attained by due managerial interventions and pieces of training of the employees.

### Limitations and Future Directions

There have been certain limitations of the study as well. (1) This study is conducted in the software industry with a technical personal; however, it can produce different results in academia and other industries. (2) This study should be conducted in other industries as well to get more generalizability of the results. (3) It is a cross-sectional study that can be conducted in the future, taking data at different points of time for more rigorous results. (4) Finally, it has taken the workplace incivility as the product of knowledge hiding; however, more variables have been proposed in the literature to be checked for better understanding.

## Data Availability Statement

The original contributions presented in the study are included in the article/supplementary material, further inquiries can be directed to the corresponding author.

## Ethics Statement

The studies involving human participants were reviewed and approved by the Renmin University of China (RUC), China. The patients/participants provided their written informed consent to participate in this study. The study was conducted in accordance with the Declaration of Helsinki.

## Author Contributions

JW conceived and designed the concept and wrote the manuscript. RM and JZ collected the data and helped in analysis. All authors read and agreed to the published version of the manuscript.

## Conflict of Interest

The authors declare that the research was conducted in the absence of any commercial or financial relationships that could be construed as a potential conflict of interest.

## Publisher’s Note

All claims expressed in this article are solely those of the authors and do not necessarily represent those of their affiliated organizations, or those of the publisher, the editors and the reviewers. Any product that may be evaluated in this article, or claim that may be made by its manufacturer, is not guaranteed or endorsed by the publisher.
